# Low urinary iodine is a protective factor of central lymph node metastasis in papillary thyroid cancer: a cross-sectional study

**DOI:** 10.1186/s12957-021-02302-6

**Published:** 2021-07-12

**Authors:** Ziyang Zeng, Kang Li, Xianze Wang, Siwen Ouyang, Zimu Zhang, Zhen Liu, Juan Sun, Xin Ye, Weiming Kang, Jianchun Yu

**Affiliations:** 1 Department of General Surgery, Peking Union Medical College Hospital, Chinese Academy of Medical Sciences and Peking Union Medical College, No. 1 Shuaifuyuan Wangfujing Dongcheng District, Beijing, 100730 China; 2 Department of Vascular Surgery, Peking Union Medical College Hospital, Chinese Academy of Medical Sciences and Peking Union Medical College, Beijing, China

**Keywords:** Iodine intake, Universal salt iodization, Papillary thyroid cancer, Papillary thyroid microcarcinoma, Central lymph node metastasis

## Abstract

**Background:**

An abrupt increase of thyroid cancer has been witnessed paralleling the supplemented iodine intake in formerly iodine-deficient countries. And increased iodine intake has been linked to the rising incidence rate of papillary thyroid cancer (PTC). However, the correlation between iodine and clinicopathological features of PTC has not been well-characterized. This study aimed to investigate the associations between iodine intake and the clinicopathological features of PTC patients.

**Methods:**

Three hundred and fifty-nine PTC patients who received surgical treatment in Peking Union Medical College Hospital from May 2015 to November 2020 were retrospectively reviewed. The associations between urinary iodine (UI), urinary iodine/creatinine ratio (UI/U-Cr), and the clinicopathological features of PTC were analyzed. Univariate and multivariate analysis were performed to investigate the relationship between UI level and central lymph node metastasis (CLNM).

**Results:**

There were no significant differences in UI in different groups according to the variables studied, except that patients with CLNM had higher UI level than CLNM(−) patients. No associations were found between UI/U-Cr and clinicopathological features except variant subtypes (classic/follicular). After dividing patients into high-iodine group and low-iodine group, more patients were found to have CLNM in the high-iodine group (p = 0.02). In addition, younger age, larger tumor size, and classic variant were positively correlated with CLNM (p < 0.05). Univariate analysis showed that insufficient iodine intake (≤ 99 μg/L) was associated with decreased CLNM risk in PTC. And after defining insufficient iodine intake as ≤ 109 μg/L and above requirements as ≥ 190 μg/L, multivariate analysis showed that lower iodine was associated with CLNM in total population of PTC (OR 0.53, 95% CI 0.31–0.91) and in PTC < 1 cm (papillary thyroid microcarcinoma, PTMC) (OR 0.43, 95% CI 0.21–0.87).

**Conclusions:**

Low iodine was a protective factor for CLNM in papillary thyroid cancer, particularly in those < 1 cm. These results indicated that iodine may not only be an initiator of tumorigenesis, but also a promoter of the development of PTC.

**Supplementary Information:**

The online version contains supplementary material available at 10.1186/s12957-021-02302-6.

## Background

Thyroid cancer is by far the most common endocrine cancer and ranked the ninth in new cancer cases globally [1]. During the past decades, thyroid cancer has been rapidly growing and is estimated to be the 4th most common cancer in 2030 globally [2]. The reason for the high increasing rate has been inconclusive over time. Risk factors have been revealed such as radiation exposure [3], industrialized lifestyle such as obesity [4], pollutants [5], and population aging as well as growing scrutiny of thyroid and improved diagnostics [6]. Apart from these known ones, iodine intake has been suggested as an important impact factor for the rise of thyroid cancer.

The thyroid plays an important role in human health, regulating metabolism, body temperature, growth, development, and organ functions. Meanwhile, iodine is the indispensable trace element for the synthesis of thyroid hormone, and deficiency of iodine can result in nodule goiter, cognitive impairments, and delayed physical development [7]. For the purpose of correcting iodine-deficient disorders, iodine prophylaxis was introduced in countries with deficient environmental iodine. However, paralleling the increase of population iodine intake, many countries reported the increase of thyroid cancer, particularly papillary thyroid cancer (PTC) [8–11]. The same trend of PTC also took place in China [12], after the universal salt iodization (USI) program was introduced in 1996. Although dietary iodine greatly improved endemic thyroid disorders, it has been debated whether over intake of iodine promoted thyroid cancer. However, because thyroid screening and imaging system were also advancing during the time, epidemiological studies were limited to reveal the true effects of increased iodine intake. As the excretion of urinary iodine accounted for 80–90% of daily iodine intake, urinary iodine is a convenient, economic and excellent biomarker to reflect the iodine intake of the population, and the World Health Organization (WHO) has recommended urinary iodine (UI), preferably median urinary iodine (MUI) to assess iodine intake [13]. Studies by Wang et al. [14] found higher UI in patients with PTC than benign nodules and found higher iodine level in LNM(+) PTC than in LNM(−) PTC. However, Zhao et al. [15] did not observe significant difference of UI between benign nodules and PTC nor did they find the difference of UI between LNM(+) and LNM(−) PTC [16]. The controversies between studies indicated that more variables should be considered in the analysis of iodine and thyroid cancer.

On the other hand, relating iodine to creatinine is yet another estimate of iodine intake, because the measurement of UI could be affected by urine output. As a result, urinary iodine/creatinine ratio (UI/U-Cr) has been established to correct the variations of urine output, because urinary creatinine excretion rate is very constant [17]. However, the majority of the studies have focused on UI, and limited studies have investigated the clinical value of UI/U-Cr in PTC.

In summary, the relationship between iodine intake and the characteristics of PTC has not been studied in detail. Also, the associations between UI/U-Cr and PTC are not well-characterized. The preliminary aim of this study is to analyze the associations between UI, UI/U-Cr, and the clinicopathological features of PTC, in order to explore the underlying effects of iodine on this rapidly growing malignancy.

## Methods

### Study population

From May 2015 to November 2020, 359 consecutive patients in Peking Union Medical College Hospital were retrospectively reviewed. Inclusion criteria were patients who received total thyroidectomy or lobectomy with central lymph node dissection; postoperatively diagnosed as papillary thyroid cancer by pathological examinations. Exclusion criteria included a previous history of thyroid operation, combination of hyperthyroidism or hypothyroidism, recent use of iodine-containing medication or reagents, administration of antithyroid drugs of thyroid hormones, abnormal kidney or liver function, and history of exposure to radiation at head and neck. Basic information of the patients was collected from the electronic medical record system, including age, sex, body mass index (BMI), and previous medical history. Postoperative pathology included pathological diagnosis, variant subtypes, foci number, tumor diameter, microscopical capsular invasion, combination of other thyroid disorders (e.g., nodular goiters), and detection of central lymph node metastasis (CLNM). This study was approved by the Ethics Committee of Peking Union Medical College Hospital (No. S-K1336). The informed consent requirement was waived because individual information in this study was not accessible.

### Surgical treatments

Thyroid surgery was performed in accordance with the management guidelines for thyroid cancer in China. Unilateral or bilateral thyroid resection was determined upon preoperative examination and intraoperative frozen sections as well as intraoperative surgical findings. Prophylactic central lymph node dissection was carried out as recommended by the Chinese guidelines. After surgery, the size, the number, and the pathological types of the foci were detected by the department of pathology. Tumor size was determined by its largest diameter. Multifocality was defined as the presence of ≥ 2 papillary thyroid cancer foci in the specimen, and in these cases, tumor size was represented by the largest one of the foci.

### Measurement of urinary iodine and urinary creatinine

The World Health Organization has recommended using median urinary iodine concentration (MUI) as a measurement for the iodine nutritional status of the population, with urinary iodine ≤ 99 μg/L, 100–199 μg/L, 200–299 μg/L, and ≥ 300 μg/L corresponding to insufficient, adequate, above requirements, and excessive iodine intake, respectively. Given that there was an intra-individual variation of 32% for urinary iodine excretion in a previous study [18], we adopted a modest modulation of the cutoff-point of WHO criteria in our analysis, where indicated. Fasting urine samples were collected during a routine visit before surgery, and the patients were required to abstain from iodine-sufficient food for at least 3 days before sample collection. On the day of sample collection, urine was collected in a clean 5-mL white-capped VACUEET tubes with no additive. Urinary iodine concentration was measured with inductively coupled plasma mass spectrometry (ICP-MS) method. The ICP-MS analysis was performed with an iCAP-Q ICP-MS system (Thermo Scientific, Waltham, MA) coupled with an automated sampler (CETAC ASX-520; Thermo Scientific, Waltham, MA) [19]. Urinary creatinine (Maccura, China, enzymatic method) was measured using an automated chemistry analyzer (Beckman Coulter AU2700, Beckman Coulter, Brea, USA). For urinary iodine, samples were measured in triplicate, and the mean value was calculated as the iodine concentration for each sample.

### Statistical analysis

Urinary iodine (UI) and urinary iodine/creatinine (UI/U-Cr) was expressed as median (upper and lower quartile) and analyzed with Wilcoxon rank-sum test because of their skewed distribution. Categorical variables were analyzed with chi-square test or Fisher’s exact test. Univariate and multivariate analyses were performed using logistic regression analysis. As UI/U-Cr was strongly associated with age and sex composition of the investigated population, propensity score matching (PSM) was applied in the analysis of UI/U-Cr to reduce the bias between groups. Age and sex were included in the algorithm using nearest-neighbor matching method with a caliper size of 0.05. The survival curve was depicted using the Kaplan-Meier method and compared using the log-rank test. A P value ≤ 0.05 was considered statistically significant. All statistical analyses were performed with SPSS software (version 26.0, IBM Corporation) and R software (version 3.6.2, R Foundation for Statistical Computing).

## Results

### UI and UI/U-Cr level in different clinicopathologic status among PTC patients

Among the 359 patients diagnosed with PTC, UI was not significantly different between different age, sex, or other clinicopathological features, except that UI was significantly higher in CLNM(+) patients than in N0 patients (152 μg/L vs 126 μg/L, p = 0.028). After correction with creatinine, female and patients aged ≥ 45 exhibited significantly higher UI/U-Cr. Except variant subtypes (p = 0.021), no other pathological features were significantly different regarding UI/U-Cr level. Of note, capsular invasion tended to correlate with lower UI, but the difference was not statistically significant (p = 0.066) (Table 1).

**Table 1:** Comparisons of UI and UI/U-Cr between different clinicopathological status of PTC patients

Variables	n	UI, μg/L	*P* value	UI/Cr, μg/g	*P* value
**Age**					
≥ 45	155	131 (87–198)	0.266	97 (75–138)	< 0.001
< 45	204	147 (97–210)		80 (56–124)	
**Sex**					
Male	104	146 (103–201)	0.353	76 (51–111)	< 0.001
Female	255	140 (86–208)		95 (70–142)	
**BMI, kg/m**^**2**^					
≥ 24	205	132 (90–197)	0.261	86 (62–124)	0.202
< 24	154	152 (96–218)		93 (67–137)	
**Tobacco use**					
Yes	38	136 (94–218)	0.782	86 (54–123)	0.15
No	321	140 (87–196)		91 (64–132)	
**Tumor size**					
≥ 1 cm	125	139 (86–197)	0.711	95 (61–132)	0.602
< 1 cm	234	142 (94–209)		87 (63–127)	
**Multifocality**					
Yes	131	148 (96–208)	0.53	95 (68–136)	0.137
No	228	136 (90–205)		86 (61–124)	
**Capsular invasion**					
Yes	253	139 (84–195)	0.066	90 (61–131)	0.65
No	106	148 (104–219)		90 (64–127)	
**Pathological subtype**					
Classic variant	261	142 (94–209)	0.484	86 (61–131)	0.021
Follicular variant	65	126 (77–197)		90 (62–124)	
Classic and follicular variant	33	166 (87–215)		102 (85–143)	
**CLNM**					
Yes	166	152 (105–215)	0.028	94 (61–141)	0.523
No	193	126 (84–197)		90 (64–123)	

### Correlations of UI, UI/U-Cr, and clinicopathological features of PTC patients

Using the median value as the cutoff, the patients were classified into an iodine-high and an iodine-low group to compare the difference between clinical and histopathological features. There was no significant difference in age, sex, BMI, smoking status, tumor size, multifocality, and capsular invasion as well as variant subtypes in the two groups. In addition, iodine was not associated with coexisted nodular goiter, chronic thyroiditis, or thyroid adenoma. However, patients in the UI-high group were more likely to be positive for CLNM compared with the UI-low group (p = 0.017) (Table 2).

**Table 2:** Association of UI and clinicopathological features of PTC

Variables	Urinary iodine, μg/L			
	n (%)	Low (%)	High (%)	*P* value
		n = 181	n = 178	
**Age**				
≥ 45	155 (43.2)	82 (22.8)	73 (20.3)	0.273
< 45	204 (56.8)	96 (26.7)	108 (30.1)	
**Sex**				
Male	104 (29)	48 (13.4)	56 (15.6)	0.407
Female	255 (71)	130 (36.2)	125 (34.8)	
**BMI, kg/m**^**2**^				
≥ 24	205 (57.1)	109 (30.4)	96 (26.7)	0.117
< 24	154 (42.9)	69 (19.2)	85 (23.7)	
**Diabetes**				
Yes	16 (4.5)	9 (2.5)	7 (1.9)	0.585
No	343 (95.5)	169 (47.1)	174 (48.5)	
**Hypertension**				
Yes	63 (17.5)	37 (10.3)	26 (7.2)	0.11
No	296 (82.5)	141 (39.3)	155 (43.2)	
**Tobacco use**				
Yes	38 (10.6)	19 (5.3)	19 (5.3)	0.957
No	321 (89.4)	159 (44.3)	162 (45.1)	
**Tumor size**				
≥ 1 cm	125 (34.8)	64 (17.8)	61 (17)	0.654
< 1 cm	234 (65.2)	114 (31.8)	120 (33.4)	
**Multifocality**				
Yes	131 (36.5)	60 (16.7)	71 (19.8)	0.277
No	228 (63.5)	118 (32.9)	110 (30.6)	
**Capsular invasion**				
Yes	253 (70.5)	129 (35.9)	124 (34.5)	0.41
No	106 (29.5)	49 (13.6)	57 (15.9)	
**Nodular goiter**				
Yes	99 (27.6)	52 (14.5)	47 (13.1)	0.491
No	260 (72.4)	126 (35.1)	134 (37.3)	
**Chronic thyroiditis**				
Yes	94 (26.2)	50 (13.9)	44 (12.3)	0.415
No	265 (73.8)	128 (35.7)	137 (38.2)	
**Thyroid adenoma**				
Yes	5 (1.4)	3 (0.8)	2 (0.6)	0.683
No	354 (98.6)	175 (48.7)	179 (49.9)	
**Variant subtype**				
Classic	261 (72.7)	127 (35.4)	134 (37.3)	0.173
Follicular	65 (18.1)	38 (10.6)	27 (7.5)	
Classic and follicular (multiple foci)	33 (9.2)	13 (3.6)	20 (5.6)	
**CLNM**				
Yes	166 (46.2)	71 (19.8)	95 (26.5)	0.017
No	193 (53.8)	107 (29.8)	86 (24)	

As is shown in Table 3, female and patients aged ≥ 45 showed significantly higher UI/U-Cr (p < 0.001 and p < 0.01), which was due to the physiologically lower U-Cr among women and elders (Table 1). To reduce the imbalance of baseline data between the two groups (Table 3), a 1:1 PSM was performed to adjust for age and sex. After PSM, 118 age- and sex-adjusted patients in each of the two groups were identified, with no statistically differences in baseline characteristics. However, no significant differences were found in histopathological features and the distribution of CLNM (p = 0.295) (Table 3).

**Table 3:** Correlation of UI/U-Cr and clinicopathological features of PTC

Variables	Before PSM				After PSM			
	n	UI/U-Cr, μg/g		*P* value	n	UI/U-Cr, μg/g		*P* value
		Low	High			Low	High	
		n = 176	n = 183			n = 118	n = 118	
**Age**								
≥ 45	155 (43.2)	60 (16.7)	95 (26.5)	0.001	105 (44.5)	49 (20.8)	56 (23.7)	0.359
< 45	204 (56.8)	116 (32.3)	88 (24.5)		131 (55.5)	69 (29.2)	62 (26.3)	
**Sex**								
Male	104 (29)	63 (17.5)	41 (11.4)	0.005	59 (25)	27 (11.4)	32 (13.6)	0.452
Female	255 (71)	113 (31.5)	142 (39.6)		177 (75)	91 (38.6)	86 (36.4)	
**BMI, kg/m**^**2**^								
≥ 24	205 (57.1)	105 (29.2)	100 (27.9)	0.337	130 (55.1)	67 (28.4)	63 (26.7)	0.601
< 24	154 (42.9)	71 (19.8)	83 (23.1)		106 (44.9)	51 (21.6)	55 (23.3)	
**Diabetes**								
Yes	16 (4.5)	3 (0.8)	13 (3.6)	0.013	10 (4.2)	2 (0.8)	8 (3.4)	0.102
No	343 (95.5)	173 (48.2)	170 (47.4)		226 (95.8)	116 (49.2)	110 (46.6)	
**Hypertension**								
Yes	63 (17.5)	29 (8.1)	34 (9.5)	0.601	41 (17.4)	19 (8.1)	22 (9.3)	0.606
No	296 (82.5)	147 (40.9)	149 (41.5)		195 (82.6)	99 (41.9)	96 (40.7)	
**Tobacco use**								
Yes	38 (10.6)	21 (5.8)	17 (4.7)	0.416	25 (10.6)	12 (5.1)	13 (5.5)	0.832
No	321 (89.4)	155 (43.2)	166 (46.2)		211 (89.4)	106 (44.9)	105 (44.5)	
**Tumor size**								
≥ 1 cm	125 (34.8)	55 (15.3)	70 (19.5)	0.164	88 (37.3)	40 (16.9)	48 (20.3)	0.282
< 1 cm	234 (65.2)	121 (33.7)	113 (31.5)		148 (62.7)	78 (33.1)	70 (29.7)	
**Multifocality**								
Yes	131 (36.5)	57 (15.9)	74 (20.6)	0.113	93 (39.4)	43 (18.2)	50 (21.2)	0.351
No	228 (63.5)	119 (33.1)	109 (30.4)		143 (60.6)	75 (31.8)	68 (28.8)	
**Capsular invasion**								
Yes	253 (70.5)	123 (34.3)	130 (36.2)	0.811	162 (68.6)	83 (35.2)	79 (33.5)	0.575
No	106 (29.5)	53 (14.8)	53 (14.8)		74 (31.4)	35 (14.8)	39 (16.5)	
**Nodular goiter**								
Yes	99 (27.6)	50 (13.9)	49 (13.6)	0.729	66 (28)	34 (14.4)	32 (13.6)	0.772
No	260 (72.4)	126 (35.1)	134 (37.3)		170 (72)	84 (35.6)	86 (36.4)	
**Chronic thyroiditis**								
Yes	94 (26.2)	40 (11.1)	54 (15)	0.144	63 (26.7)	28 (11.9)	35 (14.8)	0.303
No	265 (73.8)	136 (37.9)	129 (35.9)		173 (73.3)	90 (38.1)	83 (35.2)	
**Thyroid adenoma**								
Yes	5 (1.4)	4 (1.1)	1 (0.3)	0.207	3 (1.3)	2 (0.8)	1 (0.4)	1
No	354 (98.6)	172 (47.9)	182 (50.7)		233 (98.7)	116 (49.2)	117 (49.6)	
**Variant subtype**								
Classic	261 (72.7)	136 (37.9)	125 (34.8)	0.054	164 (69.5)	84 (35.6)	80 (33.9)	0.432
Follicular	65 (18.1)	30 (8.4)	35 (9.7)		48 (20.3)	25 (10.6)	23 (9.7)	
Classic and follicular (multiple foci)	33 (9.2)	10 (2.8)	23 (6.4)		24 (10.2)	9 (3.8)	15 (6.4)	
**CLNM**								
Yes	166 (46.2)	80 (22.3)	86 (24)	0.77	106 (44.9)	49 (20.8)	57 (24.2)	0.295
No	193 (53.8)	96 (26.7)	97 (27)		130 (55.1)	69 (29.2)	61 (25.8)	

### Risk factors of central lymph node metastasis in PTC patients

According to the results above, CLNM was closely related to a higher level of UI. However, CLNM is a pathological process affected by multiple risk factors, which could be potential confounders in the relationship of urinary iodine level and lymph node metastasis. To better understand the relationship between UI and CLNM, we further explored the risk factors that may contribute to the presence of CLNM. As Table 4 shows, age was significantly lower in patients with CLNM (p < 0.001). Tumor size was significantly larger in patients with CLNM (p < 0.001). The classic variant of PTC was more likely to present with CLNM than the follicular variant (p < 0.001). Furthermore, CLNM was marginally correlated with multiple foci (p = 0.064) (Table 4).

**Table 4:** Risk factors of central lymph node metastasis for PTC patients

Variables	CLNM		*P* value
	Yes	No	
**Age**			
≥ 45	53	102	< 0.001
< 45	113	91	
**Sex**			
Male	53	51	0.252
Female	113	142	
**BMI, kg/m**^**2**^			
≥ 24	92	113	0.551
< 24	74	80	
**Tumor size**			
≥ 1 cm	74	51	< 0.001
< 1 cm	92	142	
**Multifocality**			
Yes	69	62	0.064
No	97	131	
**Capsular invasion**			
Yes	120	133	0.484
No	46	60	
**Nodular goiter**			
Yes	41	58	0.258
No	125	135	
**Chronic thyroiditis**			
Yes	46	48	0.542
No	120	145	
**Thyroid adenoma**			
Yes	3	2	0.666
No	163	191	
**Variant subtype**			
Classic	135	126	< 0.001
Follicular	15	50	
Classic and follicular (multiple foci)	16	17	

### Univariate and multivariate logistic regression analyses on the urinary iodine level for the presence of CLMN in PTC patients

First, we investigated the association of age, tumor size, multifocality, variant subtype, and the presence of CLNM by univariate logistic regression analysis. In total PTC patients, age, tumor size, and classic variant were significantly associated with CLNM with an OR of 0.96 (95% CI 0.94–0.98), 2.01 (95% CI 1.31–3.14), and 3.57 (95% CI 1.95–6.88). Meanwhile, multifocality was marginally related to positive CLNM (OR 1.50, 95% CI 0.98–2.32).

**Fig. 1:**
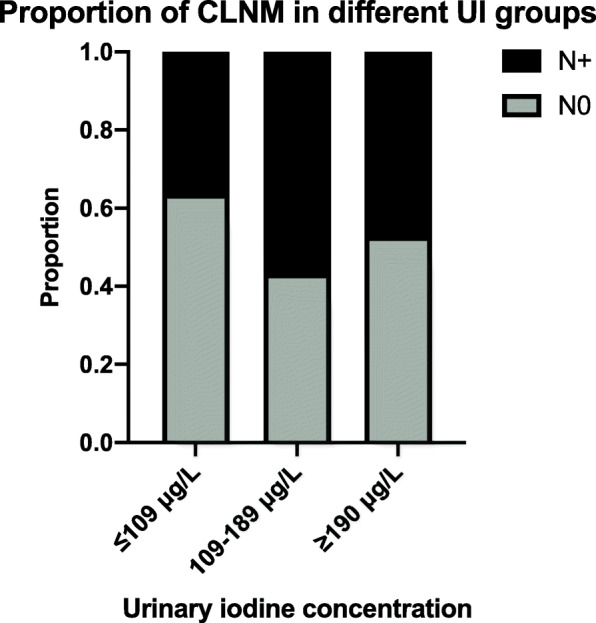
Proportion of CLNM of PTC patients with different UI levels

The optimal cutoff value of UI for the diagnosis of CLNM was determined as 130.5 μg/L (Supplementary figure 1). Then, based on the WHO criteria, we found that insufficient iodine (≤ 99 μg/L) tended to be inversely associated with CLNM by univariate regression analysis (Table 5). However, no significant associations were found after adjusted for age, sex, multifocality, tumor size, and variant subtype. After we defined insufficient iodine as ≤ 109 μg/L and above requirements as ≥ 190 μg/L, we found that low UI was a protective factor for CLNM by univariate analysis (OR 0.44, 95% CI 0.26–0.72) and remained an independent protective factor in multivariate analysis (OR 0.53, 95% CI 0.31–0.91). In subgroup analysis, lower iodine was also correlated with N0 both in PTC ≥ 1 cm and PTC < 1 cm by univariate analysis. And multivariate analysis revealed that low UI remained an independent protective factor for CLNM in PTC < 1 cm (OR 0.43, 95% CI 0.21–0.87) (Table 5). As Fig. 1 shows, PTC with ≤ 109 μg/L had the smallest proportion of CLNM, while the group with ≥ 190 μg/L and 110–189 μg/L showed medium and higher proportion of CLNM. However, the recurrence-free survival was not significantly different between the three groups (p = 0.49) (Supplementary figure 2).

**Table 5:** Univariate and multivariate logistic regression analysis on UI level for the presence of CLNM in PTC patients

Urinary iodine status (μg/L)	Univariate			Multivariate^c^		
	OR	95% CI	*P* value	OR	95% CI	*P* value
**Total patients**^**a**^						
≤ 99 (n = 102)	0.59	0.36–0.98	0.043	0.71	0.41–1.23	0.221
100–199 (n = 164)	Reference			Reference		
≥ 200 (n = 93)	0.98	0.59–1.63	0.934	0.99	0.59–1.83	0.98
**PTC ≥ 1 cm**^**a**^						
≤ 99 (n = 39)	0.4	0.17–0.91	0.031	0.51	0.20–1.30	0.161
100–199 (n = 56)	Reference			Reference		
≥ 200 (n = 30)	1.03	0.41–2.69	0.956	1.26	0.44–3.73	0.674
**PTC < 1 cm**^**a**^						
≤ 99 (n = 63)	0.7	0.36–1.33	0.281	0.73	0.35–1.48	0.381
100–199 (n = 108)	Reference			Reference		
≥ 200 (n = 63)	0.98	0.52–1.85	0.96	1.09	0.55–2.15	0.815
**Total patients**^**b**^						
≤ 109 (n = 135)	0.44	0.26–0.72	0.001	0.53	0.31–0.91	0.021
110–189 (n = 120)	Reference			Reference		
≥ 190 (n = 104)	0.68	0.40–1.15	0.154	0.71	0.41–1.29	0.281
**PTC ≥ 1 cm**^**b**^						
≤ 109 (n = 50)	0.38	0.16–0.89	0.028	0.59	0.22–1.58	0.294
110–189 (n = 42)	Reference			Reference		
≥ 190 (n = 33)	0.9	0.34–2.40	0.826	1.33	0.45–4.06	0.608
**PTC < 1 cm**^**b**^						
≤ 109 (n = 85)	0.44	0.23–0.83	0.012	0.43	0.21–0.87	0.019
110–189 (n = 78)	Reference			Reference		
≥ 190 (n = 71)	0.61	0.32–1.18	0.143	0.65	0.32–1.33	0.242

^a^Iodine insufficient, adequate, and above requirements (including excessive) were defined as ≤ 99 μg/L, 100–199 μg/L, and ≥ 200 μg/L

^b^Iodine insufficient, adequate, and above requirements (including excessive) were defined as ≤ 109 μg/L, 110–189 μg/L, and ≥ 190 μg/L

^c^Adjusted for age, sex, multifocality, tumor size, and variant subtype

## Discussion

It is well established that the association between iodine intake and the incidence rate of thyroid disorders follows a U-shaped pattern [20], which demonstrates that either higher or lower iodine is likely to cause thyroid pathogenesis. The major finding of this study is that low iodine was associated with less LNM in the clinical practice. Although some studies suggested high iodine promoted PTC development, whereas this only indicated the correlation of iodine overdose and cancer development. Few studies have investigated how iodine affects PTC in a normal range or below (UI < 200 μg/L). With the most control to biases, the current study found that iodine range below sufficient could be protective for CLNM. This finding added yet another layer of regulatory relation between iodine intake and thyroid. On the other hand, UI/U-Cr did not exhibit a strong correlation with characteristics of PTC like urinary iodine did. As an improved formula, UI/U-Cr was first introduced to normalize the differences of urine output when measuring UI. However, age, sex, and even protein intakes have strong influences on creatinine production [17, 21]. By adjusting for age and sex, we did not observe significant relevance of UI/U-Cr to the histopathology of PTC. Thus, this study indicated that there may be no apparent associations between UI/U-Cr and PTC, which is in agreement with the notion that variations of UI could be evened out among population and that relating UI to creatinine is to some extent an unnecessary procedure [13].

Wang et al. [14] found significantly higher MUI in positive vs negative lymph node metastasis (1584.62 μg/L vs 315.61 μg/L) (2010–2011). Zhao et al. [16] conducted a similar investigation and did not find difference of MUI between positive and negative CLNM. However, after defining CLNM as metastatic LN ≥ 2, they found those with CLNM(+) had a higher MUI (191.7 μg/L vs 176.2 μg/L) (2013–2018). It is possible that at different baseline levels, iodine could have different impacts on the tumor behavior. The different results of the studies could be attributed to different geographical environment, as the former was conducted in an iodine-sufficient coastal area and the latter conducted in an inland region. The present study, conducted inland, showed that the MUI of population was at a relatively low level. This may have caused the milder differences between groups in our analysis. Moreover, it should be noted that since 2012, the standard iodine concentration in iodized salt was reduced from 35 mg/kg to a range of 20 to 30 mg/kg in China. This further resulted in a decrease of MUI in the population. Nevertheless, by classifying iodine status into a higher and lower group, this study found more CLNM cases in the high-iodine group, suggesting that even within a lower range of iodine level, iodine may still have an impact on the pathological features of PTC.

As the risk of lymph node metastasis is multifactorial, distinguishing the risk factors for CLNM was necessary before we investigated the correlation of iodine and lymph node metastasis. Our study found CLNM was mostly correlated with age and tumor size, which was evidenced by previous meta-analyses [22–24]. Moreover, studies have indicated higher risk of LNM in classic PTC compared to follicular variant PTC, which resulted in poorer long term outcomes for classic PTC [25]. Thus, we have considered these important factors in the assessment of CLNM risk. Referring to the WHO criteria (≤ 99 μg/L for insufficient iodine intake, 100–199 μg/L for adequate iodine intake, and ≥ 200 μg/L for above requirements), there was a tendency that CLNM decreased with lower urinary iodine. Allowing for the natural variation of spot urinary iodine, we redefined insufficient iodine as ≤ 109 μg/L and above requirements as ≥ 190 μg/L. And we found insufficient iodine to be significantly associated with CLNM(−) for PTC < 1 cm in subgroup analysis. We thus supposed that urinary iodine may be more closely related to CLNM in PTC < 1 cm instead of PTC ≥ 1 cm. In the similar pattern, Zhao et al. [16] only observed high UI to be marginally associated with CLNM. The different observations could be explained from several aspects. First is the absence of stratification for PTC sized ≥ 1 cm and < 1 cm. As is shown in our study, iodine status may be associated with CLNM more in PTC < 1 cm than in PTC ≥ 1 cm, indicating that there are more influencing factors in PTC ≥ 1 cm. Second, we included classic and follicular variant as covariate in our model, as the classic/follicular ratio could largely affect the prevalence of CLNM. However, the classification of classic and follicular variant was often absent in previous studies. Third, we redefined and widened the range for insufficient iodine intake and above requirements, in consideration of the natural variation of urinary iodine. In clinical practice, PTC < 1 cm, or papillary thyroid microcarcinoma (PTMC) [26] are usually of good prognosis; however, 1/3 to 2/3 of PTMC could have lymph node metastasis on pathological examination [27, 28]. Therefore, it has been controversial as to whether prophylactic LN dissection should be performed on PTMC. The present study suggested that iodine should be considered a risk factor of CLNM for PTMC, and more precise stratification for iodine nutrition are needed to identify the at-risk groups for PTC patients.

In experimental researches, it was found that iodine could promote thyroid cancer growth by suppressing miR-422a and up-regulating MAPK1 [29]. Moreover, iodine may have dual roles in mediating the proliferation of thyroid cancer in vitro. At concentration below 1.0 × 10^−3^ mM it could promote cell growth and migration, while at concentration above 1.0 × 10^−3^ mM iodine exhibited an inhibitory role [30]. And within a certain range of concentration, iodine could activate Akt and Erk signaling pathway [30], which are essential for tumorigenesis, invasion, metastasis, and epithelial-mesenchymal transition [31, 32]. Furthermore, it was reported that BRAF mutation was more common in the high-water iodine region than in normal regions, while BRAF was closely related to lymph node metastases of PTC [33, 34]. These studies further confirmed iodine as a critical element during the development of thyroid cancer on the molecular basis; therefore, understanding the multi-layer mechanisms of iodine may facilitate better prevention and treatment of this malignancy.

In summary, our study showed that low UI level was a protective factor for CLNM of papillary thyroid cancer. The study also compared UI and UI/U-Cr simultaneously with the clinicopathological features of PTC and suggested that UI is a better parameter than UI/U-Cr to indicate lymph node metastasis of PTC. Our study was limited by the usual concerns related to cross-sectional studies: we cannot conclude a cause-and-effect relation between iodine and the pathological features of PTC, so further studies are still warranted to provide evidence of the regulatory mechanism between iodine and the aggressiveness of PTC. Furthermore, we did not observe significant difference of recurrence-free survival, so a longer follow up period will be required to investigate the prognosis of PTC patients with different UI levels. Although the effect of iodine on thyroid cancer is still controversial, our data further support the concept that iodine is related to lymph node metastasis of PTC, mainly through the protective effect of low iodine, which may provide novel insights into the management of iodine nutrition.

## Supplementary Information

**Additional file 1: Supplementary Figure 1.** The receiver operating characteristic (ROC) curve for the diagnosis of CLNM. The ROC yielded an area under the curve (AUC) of 0.567. The sensitivity was 0.633 and the specificity was 0.523.

**Additional file 2: Supplementary Figure 2.** Recurrence-free survival of the PTC patients with different UI levels.

## Data Availability

The datasets used and/or analyzed during the current study are available from the corresponding author on reasonable request.
