# Supplementary figures and images for: Low urinary iodine is a protective factor of central lymph node metastasis in papillary thyroid cancer: a cross-sectional study
Source: World J Surg Oncol. 2021 Jul 12;19:208. doi: 10.1186/s12957-021-02302-6 (PMC8276512; doi:10.1186/s12957-021-02302-6)

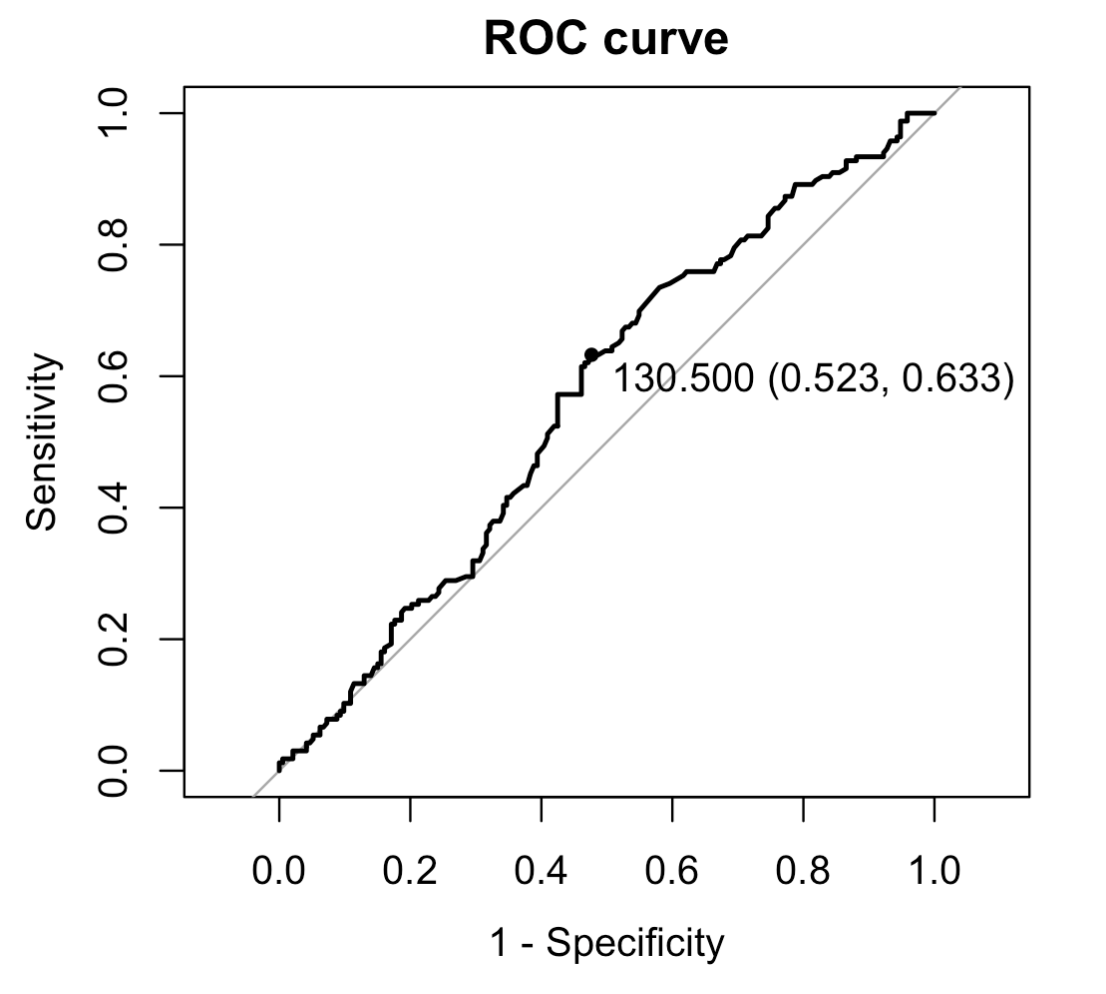

Supplement: Supplementary file 1 — Additional file 1: Supplementary Figure 1. The receiver operating characteristic (ROC) curve for the diagnosis of CLNM. The ROC yielded an area under the curve (AUC) of 0.567. The sensitivity was 0.633 and the specificity was 0.523. [file 12957_2021_2302_MOESM1_ESM.png]

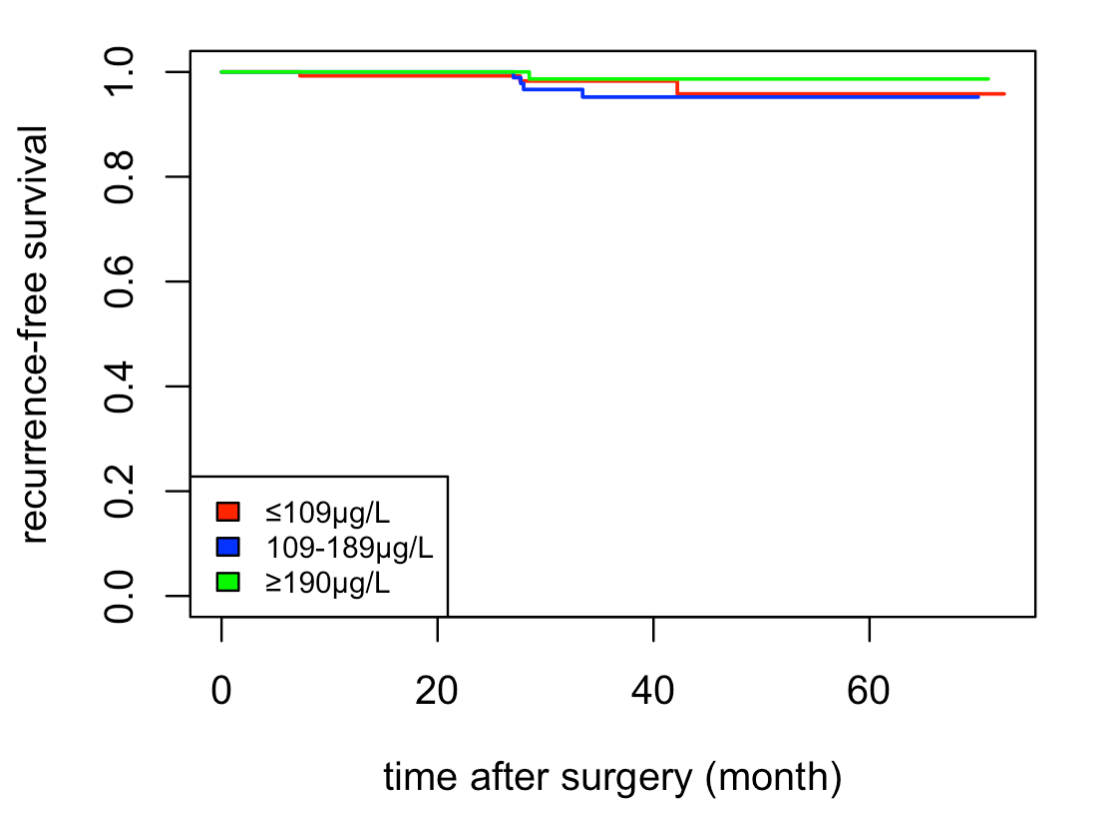

Supplement: Supplementary file 2 — Additional file 2: Supplementary Figure 2. Recurrence-free survival of the PTC patients with different UI levels. [file 12957_2021_2302_MOESM2_ESM.png]
